# Effects of Liquid Bio-Fertilizer on Plant Growth, Antioxidant Activity, and Soil Bacterial Community During Cultivation of Chinese Cabbage (*Brassica rapa* L. ssp. *pekinensis*)

**DOI:** 10.3390/microorganisms13051036

**Published:** 2025-04-30

**Authors:** Tran Yen Linh Le, Junkyung Lee, Su-Yeon Shim, Jiwon Jung, Soo-Ryang Kim, Sung-Ha Hong, Myung-Gyu Lee, Sun-Goo Hwang

**Affiliations:** 1 Department of Agricultural Convergence, Sangji University, 83 Sangjidae-gil, Wonju-si 26339, Republic of Korea; yenlinhsangji@naver.com; 2 Department of Applied Plant Science, Sangji University, 83 Sangjidae-gil, Wonju-si 26339, Republic of Korea; jklee627@naver.com (J.L.); 0108tndus@naver.com (S.-Y.S.); 3 Department of Environmental Resources, Agricultural and Rural Development, Sangji University, 83 Sangjidae-gil, Wonju-si 26339, Republic of Korea; 0728jjw@naver.com; 4 Industry-Academic Cooperation Foundation, Sangji University, 83 Sangjidae-gil, Wonju-si 26339, Republic of Korea; sooryang@daum.net (S.-R.K.); h8638669@hanmail.net (S.-H.H.); 5 Department of Smart Life Science, Sangji University, 83 Sangjidae-gil, Wonju-si 26339, Republic of Korea; mqlee@sangji.ac.kr

**Keywords:** liquid bio-fertilizer, plant growth, antioxidant, soil nutrients, soil microbial diversity

## Abstract

This study investigated the effects of liquid bio-fertilizer (LBF) on the growth, antioxidant activity, soil properties, and soil microbial composition of Chinese cabbage (*Brassica rapa* L. ssp. *pekinensis*). The LBF application significantly enhanced vegetative growth by increasing the leaf length, leaf width, fresh weight, and dry weight. Additionally, antioxidant activity increased with rises in total phenolic and flavonoid contents. However, the per-unit antioxidant concentrations decreased, likely due to rapid biomass accumulation. Soil analysis showed improvements in pH, organic matter, and available phosphorus. Microbial analysis revealed that *Acidobacteria* enrichment was associated with enhanced nutrient cycling despite reduced overall microbial diversity. Transcriptomic analysis identified 445 differentially expressed genes with upregulation in the metabolism and photosynthesis-related pathways, suggesting improved nutrient assimilation and energy production. These findings demonstrate that LBF enhances plant growth and soil fertility while influencing microbial dynamics and gene expression.

## 1. Introduction

Chemical fertilizers play a crucial role in promoting plant growth, thereby ensuring crop productivity and meeting the increasing global food demand amid shrinking arable land [1,2]. However, the continuous use of chemical fertilizers tends to reduce soil porosity, leading to compaction, a decline in soil fertility, contamination of air, water, and soil, and the depletion of essential soil nutrients and minerals, thereby posing significant environmental risks [2]. Consequently, sustainable alternatives such as liquid bio-fertilizer (LBF) have gained increasing attention.

LBFs derived from microbial inoculants, plant extracts, animal manure, and organic matter have emerged as a promising solution for enhancing soil fertility, improving crop growth, and mitigating environmental impacts [3,4,5]. These fertilizers supply essential nutrients and promote beneficial microbial activity, thereby contributing to overall soil health and crop resilience [6,7,8]. Moreover, they improve the soil’s structure and water retention and reduce nutrient leaching compared to conventional fertilizers [8,9,10,11], aligning with the principles of regenerative agriculture [12,13,14].

Chinese cabbage (*Brassica rapa* L. ssp. *pekinensis*) belongs to the Brassicaceae family and is widely consumed in Asian countries [15]. It is a rich source of dietary fiber, vitamins, minerals, and natural antioxidants, including carotenoids, flavonoids, vitamins, and various phenolic compounds [16,17,18,19]. Its antioxidant-rich profile makes it valuable for both fresh consumption and fermentation into kimchi [20,21]. The application of LBF or bio-organic fertilizers can significantly enhance the growth performance and nutritional quality of Chinese cabbage [22,23]. The integration of bio-organic fertilization has been shown to significantly enhance biyearly yields and the commercial quality of Chinese cabbage [24]. Furthermore, the use of sustainable inputs, such as microalgae-based bio-fertilizers, can improve yields while reducing dependency on conventional chemical fertilizers, potentially increasing the cost-effectiveness of large-scale production systems [25].

In our previous study, Chinese cabbage was cultivated in pots under greenhouse conditions, and the results demonstrated that LBF effectively promoted plant growth and improved crop quality [22]. However, greenhouse trials often fail to capture the full complexity of field environments, where variable climatic and soil conditions can significantly influence the efficacy of organic amendments. To address this limitation and further explore the practical potential of LBF, the present study was designed as a field trial to evaluate the effects of an organic LBF prepared by cultivating *Chlorella fusca* CHK0059 in a purified livestock manure-based medium, followed by centrifugation to obtain the supernatant. This study aimed to assess the impact of this LBF formulation on growth performance, antioxidant activity, and soil bacterial community composition in Chinese cabbage (*Brassica rapa* L. ssp. *pekinensis*) grown under field conditions. By integrating plant physiological metrics with microbial community analysis, this research provides further insight into the applicability and ecological effects of LBF in sustainable agriculture.

## 2. Materials and Methods

### 2.1. Plant Growth Conditions and Fertilizer

The experiment was conducted from July to September 2024, with average temperatures ranging from 25 to 28 °C. Seeds of Chinese cabbage (*Brassica rapa* L. ssp. *pekinensis*, cv. Cheongmyeong), obtained from Nongwoobio Seed Co., Ltd. (Seoul, Republic of Korea), were sown in an experimental field in Pyeongchang-gun, Republic of Korea. A total field area of approximately 2300 pyeong (approximately 7600 m^2^) was used, and the soil was classified as sandy loam. Seeds were directly sown in rows, spaced 40 cm apart within rows and 1 m between rows. The field was divided into two treatment groups: one irrigated with water only (control, CT) and the other treated with LBF. Although detailed replicate counts and randomization procedures were not recorded, the field was divided into clearly separated treatment zones with uniform planting, ensuring consistent exposure within each group.

Livestock manure was processed into an organic medium using a previously reported procedure [26], which served as the growth substrate for *Chlorella fusca* CHK0059. Specifically, fermented livestock manure that satisfied Livestock Manure Liquid Fertilizer Quality Certification (LFQC) standards was purified via an electrocoagulation process to remove suspended solids, thereby increasing light transmittance and transforming the solution into a bio-medium suitable for microalgal cultivation. This algal strain was provided by the National Institute of Agricultural Sciences (Wanju County, Republic of Korea). Cultivation was carried out under controlled incubator conditions at an average temperature of 28 °C until a cell density of 1.0 × 10⁷ cells/mL was reached. The incubation system used LED lighting modules (FNB-240LED; F&B Nature, Chungju, Republic of Korea) emitting alternating red and blue light under a 16 h light/8 h dark cycle. Aeration was maintained at a flow rate of 0.1 m^3^ air/m^3^ culture/min [27]. The cultured Chlorella medium was centrifuged at 12,000× *g* using a tubular continuous centrifuge (model J-1050A; Hanil Sci-Med, Daejeon, Republic of Korea) to separate the biomass and obtain the final liquid product. The electrical conductivity (EC) of the resulting fertilizer solution was adjusted to 1.5 mS/cm. During the cultivation period, the LBF-treated area was fertilized twice via sprinkler irrigation using 600 L of stock solution diluted at a 1:200 ratio. The first application was performed after the plants had reached a stable early developmental stage. The second application was conducted after a fixed interval following the first growth survey.

### 2.2. Plant Growth Survey

To compare plant growth between the two treatment areas, growth surveys were conducted at two time points, each approximately three weeks after LBF application. The first survey was conducted 54 days after planting (DAP) when visible differences between the treatment groups began to appear. The second survey was conducted approximately three weeks later to evaluate the effects of the second LBF application. We measured the leaf length, leaf width, chlorophyll content, fresh weight, and dry weight of Chinese cabbage plants in the CT and LBF treatments. Leaf length was defined as the length of the longest leaf, and leaf width as the widest part of the same leaf. The chlorophyll content was assessed using a SPAD-502 chlorophyll meter (Konica Minolta, Tokyo, Japan). Fresh weight was measured prior to drying, and the dry weight was recorded after dehydration at 60 °C for 24 h.

### 2.3. Chinese Cabbage Extract

The extract of Chinese cabbage was prepared after drying in accordance with the procedure mentioned earlier [28,29]. Dried leaf samples were subjected to extraction with 99% methanol (Daejung, Siheung, Republic of Korea) at 58 °C for 24 h using a shaking incubator (ED-SI300R; Hanyang Science Lab Co., Ltd., Seoul, Republic of Korea). The resulting extracts were centrifuged at 1300× *g* for 10 min (Allegra X-30R Centrifuge; BECKMAN COULTER, Indianapolis, IN, USA) to obtain the supernatants. These methanolic extracts of Chinese cabbage were subsequently used for antioxidant activity assays.

### 2.4. Antioxidant Activity Analysis

Antioxidant analysis was performed as previously described [28]. The Folin–Ciocalteu method was used to measure the total phenolic content (TPC) [30,31]. For the analysis of the total flavonoid content (TFC), a standard curve was generated using quercetin (Sigma-Aldrich, St. Louis, MO, USA) at 510 nm [32]. The experimental methods were slightly modified from those previously described for determining 2,2-diphenyl-1-picrylhydrazyl (DPPH) radical scavenging activity [33], nitrite radical scavenging activity [29], and 2,2′-azino-bis(3-ethylbenzothiazoline-6-sulfonic acid) (ABTS) radical scavenging activity [34]. The quantitative curve of the standard for each antioxidant activity experiment showed a suitable coefficient of determination (R^2^) ≥ 0.99 (Figure S1).

### 2.5. Analysis of the Chemical Composition of the Soil

Following the application of LBF, the soil’s chemical properties were analyzed. The soil pH and EC were determined using a 1:5 (*w*/*v*) soil-to-water suspension. Standard protocols were used to quantify total nitrogen (T-N), ammonium nitrogen (NH_4_^+^-N), nitrate nitrogen (NO_3_^−^-N), available phosphorus (P), potassium (K), sodium (Na), calcium (Ca), magnesium (Mg), cation-exchange capacity (CEC), and organic matter (OM). LBF samples were homogenized and immediately analyzed using the same parameters. The analytical procedures followed those described in a previous study [35] with modifications.

### 2.6. Soil Microbiome Analysis

Soil samples from the rhizosphere were collected immediately after harvesting. Genomic DNA was isolated using the DNeasy PowerSoil Kit (Qiagen, Hilden, Germany) in accordance with the manufacturer’s instructions. All DNA samples were stored at −80 °C until subsequent analysis. To assess the bacterial community composition, the V3–V4 hypervariable regions of the 16S rRNA gene were amplified. Library preparation was performed following the Illumina 16S Metagenomic Sequencing Library Preparation guide [36]. PCR amplification was performed as previously described [35], using Herculase II Fusion DNA Polymerase (Agilent Technologies, Santa Clara, CA, USA). Universal primers targeting the V3–V4 region used were as follows:

V3-F: 5′-TCGTCGGCAGCGTCAGATGTGTATAAGAGACAGCCTACGGGNGGCWGCAG-3′

V4-R: 5′-GTCTCGTGGGCTCGGAGATGTGTATAAGAGACAGGACTACHVGGGTATCTAATCC-3′

The resulting PCR amplicons were sequenced using an Illumina NovaSeq 6000 platform (Illumina, San Diego, CA, USA).

Raw sequence data were trimmed and quality-filtered using the Mothur software package [37]. To retain the high-quality reads specific to the V3–V4 region, sequences were filtered based on a minimum length of 441 bp and a maximum length of 466 bp. Amplicon sequence variants (ASVs) were taxonomically assigned to the most similar reference sequences using the SILVA SSU reference database. The data were subsequently analyzed using R (v.4.4.1). The phyloseq [38] and taxa [39] packages were used for taxonomic parsing, and unassigned taxa were removed by filtering the ASV count and taxonomy tables. Alpha diversity indices (e.g., the inverse Simpson, Shannon, and Simpson indices) were calculated using the Vegan package [40], and normality was examined using the Shapiro–Wilk test. One-way analysis of variance was performed, followed by Tukey’s post hoc testing (agricolae package) [41], and the results were displayed using the ggplot2 package [42]. Beta diversity was evaluated using Bray–Curtis dissimilarities, and clustering patterns were visualized by the pheatmap package [43]; color themes were applied using the RColorBrewer package [44]. Differences in the taxonomic composition were investigated using the metacoder package [45] through group comparisons and heat tree matrix methods with false discovery rate correction. Differences in individual sample counts were determined using DESeq2 [46], and the significance was confirmed at *p* < 0.05. The ggrepel package [47] was used to enhance label placement in the plots.

### 2.7. De Novo Transcriptome Analysis

Total RNA was extracted from Chinese cabbage samples subjected to CT and LBF treatment groups using a standard TRIzol-based protocol [48]. Library construction and paired-end sequencing were conducted on the Illumina NovaSeq 6000 platform. Raw sequencing reads were quality-filtered and adapter-trimmed using Trim Galore with the parameters optimized for NovaSeq data. Six paired-end sample datasets (three CTs and three LBFs) were individually processed to remove low-quality bases and adapter contamination [49]. Trimmed reads were concatenated and subjected to Trinity for de novo transcriptome assembly [50]. Assembly quality was assessed using the TrinityStats.pl utility, and the coding regions were predicted using TransDecoder [51]. The predicted peptide sequences from TransDecoder were aligned to the Arabidopsis thaliana UniProt/SwissProt database using BLASTP to annotate the most likely protein functions [52]. To remove redundant sequences, CD-HIT was used at a 99% identity threshold. Transcript abundance was estimated using RSEM integrated with the Bowtie2 alignments through Trinity’s align_and_estimate_abundance.pl script [53]. Quantification files were converted into a count matrix using Trinity’s abundance_estimates_to_matrix.pl. Differential gene expression between the control and treatment groups was analyzed using the DESeq2 with the run_DE_analysis.pl script [46]. Expression values were normalized by TMM and FPKM using Trinity’s run_TMM_normalization_write_FPKM_matrix.pl. A threshold of fold change >1 and adjusted *p*-value < 0.05 were applied for significance using analyze_diff_expr.pl.

Gene Set Enrichment Analysis (GSEA) was performed using the R package clusterProfiler [54] to identify functionally enriched gene sets among differentially expressed genes. For gene ontology (GO) analysis, the org.At.tair.db annotation package was used to map gene symbols to the GO terms for Arabidopsis thaliana [55]. GO enrichment analyses were conducted for the Cellular Component (CC) and Biological Process (BP) categories using the gseGO function in clusterProfiler [54].

### 2.8. Statistical Analysis

To evaluate the effects of LBF treatment on Chinese cabbage, a two-tailed Student’s *t*-test was applied for statistical analysis of all experimental data to determine the significant differences between the CT and LBF-treated groups. Data are presented as the mean ± standard deviation based on three distinct biological replicates. For quantitative assays such as antioxidant measurements and chemical component analyses, standard curves were generated, and their respective regression equations and R^2^ values were calculated using Microsoft Excel 2016. Pearson’s correlation analysis was conducted using n−2 degrees of freedom, and adjustments for multiple comparisons were made using the Benjamini–Hochberg false discovery rate method.

## 3. Results

### 3.1. Growth of Chinese Cabbage Under Different Treatments

**Figure 1:**
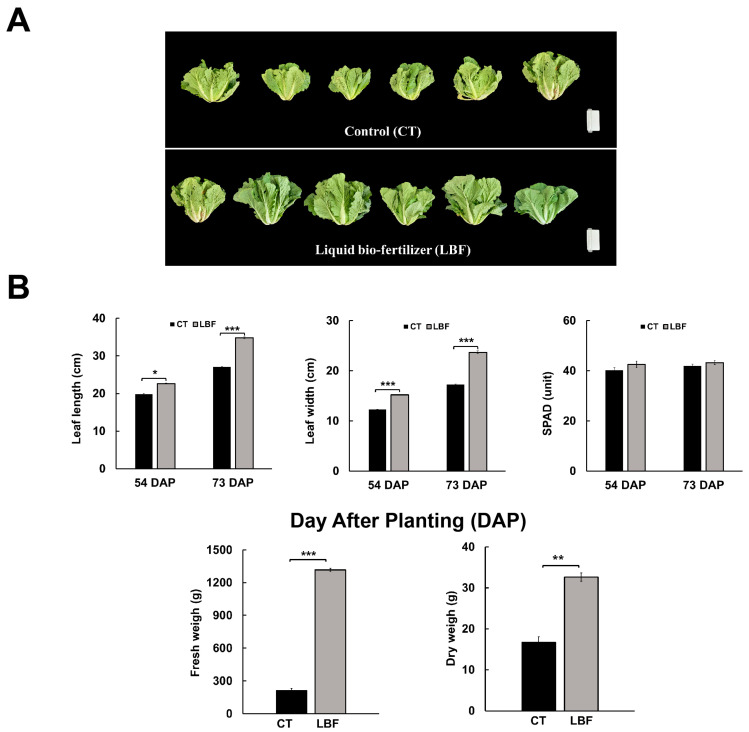
Growth performance of Chinese cabbage under different treatments (control [CT] and liquid bio-fertilizer [LBF]). (**A**) Phenotypes of Chinese cabbage. (**B**) Comparative analysis of growth parameters, including leaf length, leaf width, chlorophyll content (SPAD value), fresh weight, and dry weight. Values are presented as mean ± standard deviation (n = 9). * *p* < 0.05; ** *p* < 0.01; *** *p* < 0.001 (Student’s *t*-test).

To evaluate the growth-promoting effects of LBF, phenotypic changes in Chinese cabbage were observed on 54 and 73 DAP (Figure 1). On 73 DAP, the LBF group exhibited significant morphological improvements in leaf development and overall plant size compared to the control (CT) group, suggesting enhanced growth owing to the LBF application (Figure 1A). The leaf length, leaf width, SPAD unit, and weight of the aerial part were measured on 54 and 73 DAP (Figure 1B). On 54 DAP, the leaf length in the LBF group was 22.67 cm, which was 2.86 cm longer than that in the CT group (19.81 cm). Similarly, the leaf width in the LBF group was 15.19 cm, which was 2.92 cm wider than that in the CT group (12.27 cm). On 73 DAP, the leaf length was 34.80 cm in the LBF group and 27.08 cm in the CT group, and the leaf width was 23.68 cm in the LBF group and 17.25 cm in the CT group. The length and width of leaves increased by 1.3-fold compared to the CT group, indicating a significant enhancement in vegetative growth. The fresh weight of the LBF group was 1316.67 g, which was 6.27 times higher than that of the CT group (210 g). Similarly, the dry weight of the LBF group (49.33 g) was 2.96 times higher than that of the CT group (16.67 g). Although the SPAD index, which is an indicator of chlorophyll content, was relatively high in the LBF-treated group, the difference between the two treatment groups was not significant (*p* > 0.05). The SPAD units in the LBF group were 42.51 and 43.26 on 54 and 73 DAP, respectively, whereas the CT group showed SPAD values of 40.15 and 41.86 on 54 and 73 DAP, respectively. Overall, these results demonstrate that an LBF application significantly promotes plant growth.

### 3.2. Antioxidant Activity

**Figure 2:**
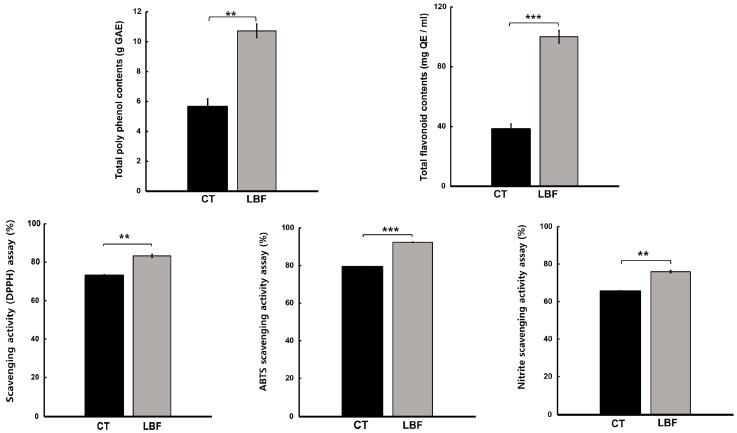
Antioxidant activity in the aerial parts of Chinese cabbage under different treatments (control [CT] and liquid bio-fertilizer [LBF]). Values are presented as mean ± standard deviation (n = 3). * *p* < 0.05; ** *p* < 0.01; *** *p* < 0.001 (Student’s *t*-test).

To investigate the effects of LBF on plant quality, the antioxidant activities, including TPC, TFC, DPPH radical, ABTS, and nitrite scavenging activities, were evaluated in the entire aerial part of Chinese cabbage after harvesting (Figure 2). The TPC values were 10.72 and 5.64 g GAE, and the TFC values were 100.06 and 38.27 g QE in the LBF and CT groups, respectively. DPPH scavenging activities were 83.32% and 73.09%, ABTS scavenging activities were 92.38% and 79.31%, and nitrite scavenging activities were 75.97% and 65.54% in the LBF and CT groups, respectively. The antioxidant activities increased in the entire aerial part of the Chinese cabbage. However, the levels of antioxidants in the same amount of plant tissues significantly decreased (Figure S2). These results suggest that LBF has a greater effect on promoting plant growth than on the accumulation of antioxidant compounds in plant cells.

### 3.3. Soil Chemical Composition

The chemical composition of the soil in each experimental field was analyzed after harvest. The application of LBF had a significant impact on several soil indicators related to nutrient availability (Table 1). The soil pH for the LBF group (5.83) was significantly higher than that for the CT group (5.44), indicating an improvement in the regulation of soil acidity. Meanwhile, EC showed a slight increase in the LBF-treated soil (3.82 dS/m) compared to that in the CT soil (3.60 dS/m); however, this difference was not significant. The organic matter content in the soil was 5.64% in the LBF group and 4.35% in the CT group, indicating that the application of LBF significantly increased the organic matter content. Furthermore, the T-N content, which includes NH_4_^+^-N and NO_3_^−^-N, was significantly higher in the LBF group than in the CT group. The LBF group had 2470.56 mg/kg T-N, 196.78 mg/kg NH_4_^+^-N, and 383.58 mg/kg of NO_3_^−^-N, whereas the CT group had 2171.28 mg/kg T-N, 78.75 mg/kg NH_4_^+^-N, and 260.88 mg/kg NO_3_^−^-N. The available P content was substantially higher in the LBF-treated soil (2644.75 mg/kg) than in the CT soil (2110.30 mg/kg), suggesting that the application of LBF significantly enhanced phosphorus availability. Finally, significant differences were observed in the exchangeable cations. The LBF group exhibited significantly higher concentrations of exchangeable Ca (8.97 cmol/kg), Mg (1.86 cmol/kg), and Na (0.30 cmol/kg) than did the CT group (6.90 cmol/kg Ca, 1.50 cmol/kg Mg, and 0.15 cmol/kg Na). The cation-exchange capacity (CEC) of the LBF group (15.5 cmol/kg) was slightly higher than that of the CT group (14.73 cmol/kg in CEC); however, the difference was not significant.

Pearson’s correlation coefficients (PCC) were analyzed to investigate the relationship between soil chemical properties (Table 2). Significant correlations were observed among the experimental fields for each chemical composition. For instance, the pH showed a significant correlation with NH_4_^+^-N, K, and Na, while CEC significantly correlated with T-N and Na. Particularly, OM exhibited a significantly positive correlation with NH_4_^+^-N, NO_3_^−^-N, available P, Ca, and Mg, indicating a strong relationship between OM and nutrient availability. Overall, the application of LBF resulted in increases in various soil chemical components, except for EC and CEC, suggesting that these improvements were associated with the accumulation of organic matter.

### 3.4. Soil Microorganism

**Figure 3:**
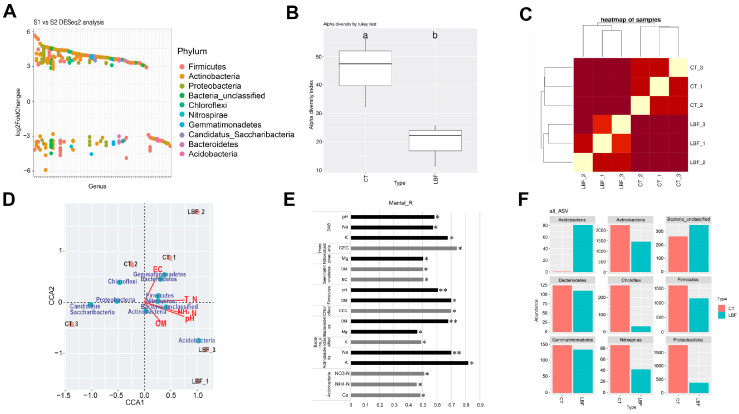
Amplicon sequence variant (ASV)-based analysis of bacterial communities in the rhizosphere of Chinese cabbage under different treatments (control [CT] and liquid bio-fertilizer [LBF]). (**A**) Differentially abundant ASVs identified between the CT and LBF groups. (**B**) Alpha diversity of the soil bacterial communities measured using the inverse Simpson index. The lowercase letters represent the significant differences (*p* < 0.05) between groups, Tukey’s HSD test. (**C**) Hierarchical clustering of samples based on Bray–Curtis dissimilarity, illustrating beta diversity patterns between the CT and LBF soils. (**D**) Canonical correspondence analysis (CCA) illustrating the correlation between bacterial communities and soil chemical properties. (**E**) Mantel test results showing significant correlations between the bacterial phyla and soil chemical properties. Values are presented as mean ± standard deviation (n = 3). * *p* < 0.05; ** *p* < 0.01; *** *p* < 0.001. (**F**) Relative abundance of the major bacterial phyla in CT and LBF soils. Colored boxes represent the relative abundances of the major bacterial phyla in each treatment group.

ASVs were analyzed to evaluate the impact of LBF on the soil microbiota (Figure 3). A total of 240 ASVs (*p* < 0.05) were identified between the CT and LBF samples (Figure 3A). These ASVs were classified into ten bacterial phyla: *Firmicutes*, *Actinobacteria*, *Proteobacteria*, *unclassified Bacteria*, *Chloroflexi*, *Nitrospirae*, *Gemmatimonadetes*, *Candidatus_Saccharibacteria*, *Bacteroidetes*, and *Acidobacteria*. Additionally, the assessment of soil microbial diversity (Alpha Diversity) revealed that the CT soil sample exhibited higher bacterial diversity than did the LBF sample, indicating the potential influence of LBF treatment on soil microbial abundance (Figure 3B). Hierarchical clustering was performed to evaluate the similarities between microbial communities in the soil samples (Figure 3C). The CT and LBF groups clustered separately on the heatmap, indicating a clear difference in microbial composition between the two experimental groups. The LBF group was positioned in the upper and lower right quadrants of the canonical correspondence analysis (CCA) plot (Figure 3D). Specifically, two samples (LBF_1 and LBF_3) of the LBF group showed a strong correlation with NH_4_^+^-N, pH, and OM among the chemical properties and with *Acidobacteria* among the bacterial phyla. This suggests that LBF application may affect several nutrient-related chemical properties by increasing the abundance of *Acidobacteria*. To demonstrate the significant correlations between the microbial communities and soil properties, a Mantel test was conducted between the bacterial phyla of the 240 ASVs and chemical properties (Figure 3E). DAB (differentially abundant bacteria) exhibited robust correlations with K (*p* < 0.05), Na (*p* < 0.05), and pH (*p* < 0.01), indicating that the soil chemical compositions significantly influenced the distinct microbial community structures across treatments. *Proteobacteria* demonstrated a strong positive correlation with CEC (*p* < 0.05), which may reflect their adaptability to soils with higher nutrient retention capacity. *Chloroflexi* showed a significant correlation with CEC (*p* < 0.05), while *Gemmatimonadetes* exhibited moderate associations with both OM (*p* < 0.05) and EC (*p* < 0.05). *Firmicutes* showed significant positive correlations with OM (*p* < 0.05) and pH (*p* < 0.05), suggesting their sensitivity to soil carbon and acidity levels. *Bacteroidetes* were positively correlated with OM (*p* < 0.01) and Mg (*p* < 0.05), suggesting their role in organic matter decomposition and mineral cycling. *Actinobacteria* were strongly associated with K (*p* < 0.05) and Na (*p* < 0.01), implying their potential salt tolerance and nutrient responsiveness. *Nitrospirae* were significantly associated with Mg (*p* < 0.05), while *Acidobacteria* exhibited correlations with multiple nitrogen-related parameters, including NH_4_^+^-N (*p* < 0.05), NO_3_^−^-N (*p* < 0.05), and Ca (*p* < 0.05), suggesting their involvement in nitrogen cycling and calcium dynamics. The relative abundance of the major bacterial phyla significantly varied between the CT and LBF-treated soils (Figure 3F). The application of LBF led to a substantial increase in *Acidobacteria* and unclassified bacteria, whereas the abundance of *Chloroflexi*, *Firmicutes,* and *Proteobacteria* decreased. However, the abundance of *Bacteroidetes* did not show any significant differences among the experimental groups. These results suggest that the application of LBF influences nutrient availability and microbial interactions.

### 3.5. De Novo Transcriptome Analysis

**Figure 4:**
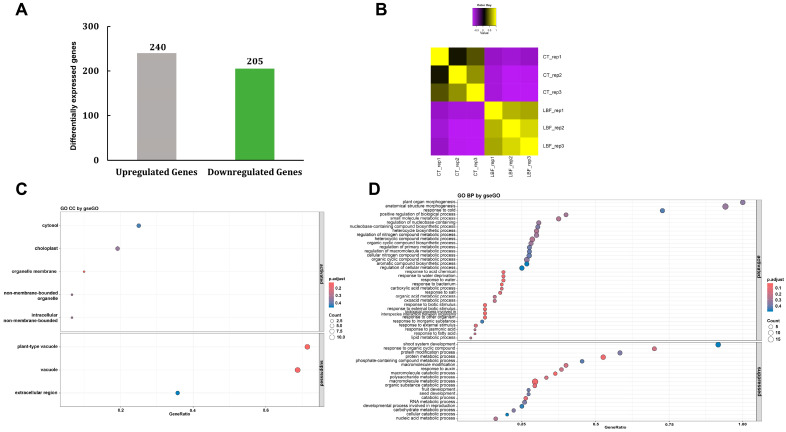
Transcriptome analysis of Chinese cabbage under control (CT) and liquid bio-fertilizer (LBF) treatments. (**A**) Number of differentially expressed genes between CT and LBF groups. Gray and green bars represent upregulated and downregulated genes. (**B**) Sample correlation matrix between CT and LBF groups. Color key indicates Pearson correlation coefficients between samples, ranging from −0.5 (purple, negative correlation) to 1 (yellow, strong positive correlation), with black representing zero or no correlation. (**C**) Cellular component (CC) category. (**D**) Biological process (BP) category. Activated terms indicate functions enriched in upregulated genes, whereas suppressed terms represent those enriched in downregulated genes. Color represents the adjusted *p*-value (p.adjust), ranging from red (more significant) to blue (less significant). Bubble size reflects the number of genes (count) associated with each GO term.

RNA-seq analysis was employed to assess the transcriptomic alterations in cabbage subjected to LBF application (Figure 4). A total of 445 genes exhibited significant differential expression genes (DEGs) (*p* < 0.05), comprising 240 upregulated and 205 downregulated genes (Figure 4A). To assess the overall similarity and clustering among samples, a sample correlation matrix was constructed based on global gene expression profiles and visualized as a heatmap (Figure 4B). The heatmap revealed a clear separation between CT and LBF, with high within-group correlation and consistent expression patterns among replicates, whereas substantial differences were observed between the two experimental groups.

GO analysis with the CC category revealed significant reorganization of subcellular structures in response to LBF treatment. The upregulated DEGs were enriched in the genes associated with organelle membranes, the cytosol, chloroplasts, and non-membrane-bounded organelles (ribosomes and protein complexes) (Figure 4C). Vacuole-related genes, particularly those involved in nutrient storage and detoxification, were downregulated. GO analysis with the BP category revealed substantial shifts in gene expression, with notable enrichment in the pathways linked to metabolism, biosynthesis of organic compounds, and photosynthesis (Figure 4D). These findings indicate that LBF may alter resource allocation to favor active growth and environmental responsiveness, potentially through hormonal regulation or adjustments of energy metabolism. The GO BP analysis further confirmed significant transcriptional shifts, highlighting the upregulation of metabolic pathways, biosynthesis of organic compounds, and photosynthesis-related processes. Genes involved in small molecule metabolism and responses to inorganic substances were significantly enriched, suggesting that LBF enhanced nutrient assimilation and biochemical activity, probably contributing to increased growth efficiency and metabolic adaptation. In contrast, suppressed biological processes were predominantly associated with lipid metabolism, carbohydrate metabolism, and macromolecular catabolism, suggesting deprioritization of energy storage in favor of active metabolic processes. Additionally, seed and shoot system development and RNA metabolic regulatory pathways were downregulated.

## 4. Discussion

Our results demonstrated significant improvements in the vegetative growth of Chinese cabbage following the application of LBF, as indicated by the increased leaf length, leaf width, and aerial biomass. These findings align with those of previous studies, which have highlighted the beneficial effects of organic biofertilizers on crop growth [56,57]. For instance, the application of a bio-organic fertilizer combined with biochar significantly promoted the growth and yield of Chinese small cabbage, attributing these enhancements to improved soil properties and nutrient availability [58]. Similarly, an organic fertilizer integrated with dried food waste powder had a positive effect on the early growth of Chinese cabbage seedlings, suggesting that the incorporation of organic waste materials into fertilizers can benefit plant development [59]. The significantly higher fresh-to-dry weight ratio observed in LBF-treated plants suggests improved water retention. Organic amendments are known to enhance the soil structure, which increases water availability to the roots and supports cellular water retention [9,60]. This enhanced water retention may be beneficial under fluctuating environmental conditions, enabling plants to maintain growth and productivity despite potential water stress. The growth outcomes observed in this field experiment are consistent with those of our previous pot-based study [22], where LBF treatment notably improved the morphological and physiological characteristics of Chinese cabbage. In that study, although LBF-induced growth was slightly lower than chemical liquid fertilizer (CLF), it outperformed fermented liquid fertilizer (FLM), indicating its potential to serve as an alternative to CLF under greenhouse conditions. Collectively, these studies reinforce the observed positive effects of LBF on Chinese cabbage growth.

The increased levels of TPC, TFC, and radical scavenging activities observed in this study indicate that LBF application positively influences the antioxidant profile of Chinese cabbage. These findings are consistent with our previous pot-based experiment [22], where LBF significantly enhanced the sugar and ascorbic acid contents, as well as key antioxidant markers such as TPC, TFC, DPPH radical scavenging activity, and FRAP. In the current field trial, LBF treatment similarly led to marked improvements in antioxidant capacity compared to water control. Although the absolute values differed between the two experiments due to environmental and methodological variations, the overall trend remained consistent.

The observed increases in TPC and TFC following LBF treatment also align with previous reports that organic fertilizers can stimulate the synthesis of secondary metabolites by improving nutrient availability [61,62]. These findings suggest that organic fertilization plays a significant role in enhancing the accumulation of phenolic and flavonoid compounds in Chinese cabbage. Notable phenolic and flavonoid constituents in Chinese cabbage, including caffeic acid, sinapic acid, p-coumaric acid, ferulic acid, and myricetin, are known for their strong antioxidant properties. These compounds contribute to the plant’s resistance to oxidative stress and may also play a protective role in human health [20]. The increased phenolic content observed in this study can be attributed to the ability of organic amendments to modulate secondary metabolism and promote antioxidant synthesis [63]. For example, phenolic-enriched organic amendments have been shown to enhance antioxidant activity in maize by stimulating phenylalanine ammonia-lyase activity, which leads to elevated levels of phenols and flavonoids [64]. In particular, the observed increase in radical scavenging activities supports prior findings that bio-fertilization enhances the antioxidant defense system by upregulating both enzymatic and non-enzymatic antioxidant components [65,66,67]. Although LBF-treated plants exhibited relatively high absolute levels of antioxidant activity, the lower concentration per unit of dry weight suggests that rapid vegetative growth may have caused a relative dilution of these metabolites. This phenomenon has been reported in previous studies, where enhanced biomass production under optimal nutrient conditions resulted in lower antioxidant concentrations per gram of tissue [68]. This balance between plant growth and secondary metabolite accumulation is a well-documented physiological response wherein plants prioritize biomass expansion over metabolite synthesis under favorable conditions [69].

The application of LBF led to a significant enrichment in soil chemical composition, with notable increases in soil pH, organic matter content, total nitrogen, and available phosphorus. This improvement is consistent with previous studies reporting that biofertilizers enhance soil quality by increasing organic matter and facilitating the availability and uptake of essential nutrients, partly through bacterial secretion and pH modulation [70,71]. Additionally, the relatively high organic matter content in LBF-treated soil contributes to improved soil structure and water retention, both of which are critical for nutrient retention and stable plant growth [72]. Organic amendments significantly enhance soil organic carbon and nutrient cycling, thereby promoting long-term soil sustainability [73]. Consistent with these findings, bio-organic fertilizers have been shown to increase organic matter and essential nutrient levels in soil compared to conventional fertilizers [74]. Moreover, the significant increase in exchangeable cations such as calcium, magnesium, and sodium suggests that LBF enhances cation-exchange capacity, which is vital for soil fertility and nutrient retention [75]. In our previous study [22], LBF application led to moderate increases in soil pH and NO_3_^−^-N levels, along with elevated exchangeable Na compared to CLF. Although total nitrogen (T-N), available phosphorous (P), organic matter (OM), and electrical conductivity (EC) did not differ significantly among treatments, measurable shifts in ammonium and nitrate concentrations indicated that different fertilizer types altered the soil nutrient profile in distinct ways, particularly in terms of nitrogen forms and pH levels.

Based on the ASV analysis, the observed shifts in microbial diversity and composition following LBF application suggest that the treatment altered the soil environment to favor specific bacterial groups while reducing overall microbial diversity. The decrease in alpha diversity in LBF-treated soil may result from microbial enrichment, where certain taxa, such as *Acidobacteria*, gain competitive advantages. Biofertilizers containing *Bacillus licheniformis* and *Bacillus amyloliquefaciens* have been shown to improve the soil’s quality by increasing the organic matter, total nitrogen, total phosphorus, and available potassium [76]. These improvements support the growth of beneficial groups such as *Proteobacteria*, *Acidobacteria*, and *Actinobacteria*. However, treatments combining multiple *Bacillus* strains have also been associated with reduced microbial diversity due to the competitive exclusion of native species. Notably, *Acidobacteria* is a group increasingly recognized for its functional diversity and ecological versatility [77]. Its enrichment under LBF treatment is particularly significant, as it plays a central role in nutrient cycling, including nitrogen transformation, organic matter decomposition, and phosphorus solubilization, especially in nutrient-rich environments. Therefore, the increase in *Acidobacteria* may signal a shift toward a more functionally active microbial community despite a reduction in taxonomic diversity. This functional restructuring does not necessarily reflect a loss in soil quality; instead, it may represent an adaptive selection for microbial groups better suited to process organic inputs introduced through LBF, thereby enhancing nutrient cycling, carbon turnover, and microbial metabolic efficiency [78].

The effects of LBF on soil bacterial communities were evident in both pot and field experiments, although the direction and extent of changes differed. In the pot study [22], LBF-treated soils exhibited higher alpha diversity than the untreated control, along with the enrichment of several genera within the *Actinobacteria* and *Proteobacteria* phyla. A key distinction between the two trials lies in the control soil conditions. In the pot experiment, the control corresponded to unplanted, untreated soil before any fertilizer application. In contrast, the field control consisted of soil collected after a full growing season under water-only irrigation. This difference likely contributed to the contrasting diversity outcomes observed across the two studies. Nevertheless, LBF application consistently induced notable shifts in microbial composition, both taxonomically and functionally. Collectively, these findings suggest that although specific microbial responses to LBF may vary depending on the initial soil conditions and plant-associated influences, the fertilizer consistently promotes ecologically relevant changes in soil bacterial communities across cultivation environments.

The RNA-seq analysis indicated transcriptional changes in the genes associated with metabolism, biosynthesis of organic compounds, and photosynthesis in Chinese cabbage following LBF application. Biofertilizers, through the activity of plant growth-promoting rhizobacteria (PGPR), enhance key metabolic pathways in plants by improving carbon fixation, nitrogen assimilation, and nutrient availability, ultimately leading to increased biomass accumulation and overall plant growth [79]. Furthermore, several photosynthesis-related genes were significantly upregulated, suggesting enhanced carbon assimilation and chlorophyll production. Similarly, the application of biofertilizers has been shown to increase the expression of genes related to the photosystem and carbon fixation pathways, resulting in improved photosynthetic efficiency [80]. The upregulation of these genes may have contributed to the relatively high biomass accumulation and leaf expansion observed in LBF-treated plants.

Following LBF application, the downregulation of genes associated with shoot system development, organic cyclic compound metabolic processes, macromolecule metabolism, fruit development, seed development, reproductive development, and carbohydrate metabolism was observed. These genes are known to be involved in fundamental processes related to plant growth, reproductive organ development, metabolic regulation, and defense responses, including antioxidative mechanisms. The suppression of genes related to carbohydrate metabolism may be particularly significant, as this pathway not only provides energy for growth but also supplies precursors for the biosynthesis of antioxidant compounds such as ascorbic acid and glutathione [81,82]. A reduced capacity for carbohydrate metabolism likely results in a decreased production of these molecules, thereby lowering the plant’s overall antioxidant potential.

Moreover, the downregulation of macromolecule metabolic processes can limit the synthesis of secondary metabolites, such as polyphenols and flavonoids, which serve as major antioxidants in plant tissues [83]. As these metabolites are often upregulated in response to environmental stresses, their suppression may reflect a shift in metabolic priorities or a reduction in stress perception under LBF treatment [84]. The reduced expression of genes related to shoot and reproductive organ development may indicate that the plant reallocated energy and metabolic resources toward maintenance rather than growth or reproduction. This shift in developmental programming could represent an adaptive response to the altered nutrient environment provided by LBF, as has been observed under various abiotic conditions [85,86].

Importantly, carbohydrate metabolism is tightly linked to the shikimate and phenylpropanoid pathways, both of which are responsible for the production of key antioxidant compounds. Therefore, the inhibition of carbohydrate-related gene expression likely contributed to the reduced biosynthesis of antioxidant metabolites, as evidenced by lower levels of total antioxidant capacity and related indices in LBF-treated plants [87,88]. Additionally, the downregulation of fruit and seed development genes may reduce the cellular demand for reactive oxygen species (ROS) regulation during reproductive organ formation. Given that ROS are naturally generated during active cell division and differentiation, particularly in developing reproductive tissues, a decrease in such activities may correspond with a reduced need for antioxidant defense systems [89].

These findings suggest that LBF treatment suppressed gene expression in key developmental and metabolic pathways, potentially reducing the plant’s requirement for antioxidant metabolism and ultimately resulting in a measurable decrease in antioxidant activity in plant tissues.

## 5. Conclusions

Our findings highlight the potential of LBF as an effective organic amendment for improving plant growth, enhancing antioxidant profiles, and enriching soil health by modifying chemical properties and microbial communities. These results support the use of LBF as a sustainable alternative to chemical fertilizers in agricultural systems. Future research should investigate the long-term effects of LBF application across various crops and environmental conditions, as well as its role in shaping soil microbial functions and plant metabolic pathways.

## Figures and Tables

**Table 1:** Chemical components of fertilizers in the soil after harvest.

Unit	CT	LBF	*p*-Value
pH [1:5]	5.44	5.83	0.011 *
EC [1:1] (dS/m)	3.60	3.82	0.225
OM (%)	4.53	5.64	0.031 *
T-N(mg/kg)	2171.28	2470.56	0.036 *
NH_4_^+^-N (mg/kg)	78.75	196.78	0.002 **
NO_3_^−^-N (mg/kg)	260.88	383.58	0.017 **
Available P (mg/kg)	2110.30	2644.75	0.002 **
Exchangeable K (cmol^+^/kg)	0.70	0.89	0.057
Exchangeable Ca (cmol^+^/kg)	6.90	8.97	0.014 *
Exchangeable Mg (cmol^+^/kg)	1.50	1.86	0.036 *
Exchangeable Na (cmol^+^/kg)	0.15	0.30	0.004 **
CEC (cmol^+^/kg)	14.74	15.50	0.051

CT, control; LBF, liquid bio-fertilizer. Values are presented as mean ± standard deviation (n = 3). * *p* < 0.05; ** *p* < 0.01; *** *p* < 0.001 (Student’s *t*-test).

**Table 2:** Pearson’s correlation among soil chemical properties.

	pH	EC	OM	T-N	NH_4_^+^-N	NO_3_^−^-N	Available P	Exchangeable K	Exchangeable Ca	Exchangeable Mg	Exchangeable Na
EC	0.069										
OM	0.766	0.232									
T-N	0.675	0.655	0.74								
NH_4_^+^-N	0.85 *	0.437	0.9 *	0.938 **							
NO_3_^−^-N	0.737	0.583	0.833 *	0.967 **	0.973						
Available P	0.794	0.364	0.889 *	0.817 *	0.934 **	0.936 **					
Exchangeable K	0.832 *	−0.083	0.476	0.463	0.63	0.581	0.72				
Exchangeable Ca	0.783	0.417	0.894 *	0.945 **	0.986 ***	0.965 **	0.903 *	0.556			
Exchangeable Mg	0.668	0.335	0.938 **	0.84 *	0.922 **	0.914 *	0.922 **	0.483	0.951 **		
Exchangeable Na	0.878 *	0.228	0.685	0.674	0.816 *	0.797	0.9 *	0.925 **	0.735	0.68	
CEC	0.718	0.532	0.428	0.821 *	0.766	0.805	0.706	0.743	0.714	0.531	0.818 *

CT, control; LBF, liquid bio-fertilizer. The fold change between the LBF and CT groups was calculated as log_2_(LBF/CT) using DESeq2. Pearson’s correlation coefficient (PCC) was evaluated for statistical significance based on n-2 degrees of freedom. * *p* < 0.05; ** *p* < 0.01; *** *p* < 0.001.

## Data Availability

The original contributions presented in this study are included in the article/Supplementary Materials. Further inquiries can be directed to the corresponding author.

## Supplementary Materials

The following supporting information can be downloaded at: https://www.mdpi.com/article/10.3390/microorganisms13051036/s1. Supplementary Figure S1. The standard curves of total phenol, total flavonoid, DPPH, and ABTS; Supplementary Figure S2. Concentration of antioxidant compounds per equal mass of aerial tissue in Chinese cabbage un-der different treatment (control [CT] and liquid bio-fertilizer [LBF]). Data are presented as mean ± standard deviation (n = 3). * *p* < 0.05; ** *p* < 0.01; *** *p* < 0.001 (Student’s *t*-test).

## Abbreviations

The following abbreviations are used in this manuscript:

LBF	Liquid bio-fertilizer
CT	Control
PGPR	Plant growth-promoting rhizobacteria
DAP	Day after planting
TPC	Total phenolic content
TFC	Total flavonoid content
DPPH	Determining 2,2-diphenyl-1-picrylhydrazyl
ABTS	2,2′-azino-bis(3-ethylbenzothiazoline-6-sulfonic acid)
EC	Electrical conductivity
T-N	Total nitrogen
NH_4_^+^-N	Ammonium nitrogen
NO_3_^−^-N	Nitrate nitrogen
P	Phosphorus
K	Potassium
Na	Sodium
Ca	Calcium
Mg	Magnesium
OM	Organic matter
CEC	Cation exchange capacity
PCC	Pearson’s correlation coefficients
ASVs	Amplicon sequence variants
CCA	Canonical correspondence analysis
GO	Gene ontology
CC	Cellular component
BP	Biological process
DEGs	Differentially expressed genes
DAB	Differentially abundant bacteria
