# Supplementary figures and images for: Effects of Liquid Bio-Fertilizer on Plant Growth, Antioxidant Activity, and Soil Bacterial Community During Cultivation of Chinese Cabbage (Brassica rapa L. ssp. pekinensis)
Source: Microorganisms. 2025 Apr 30;13(5):1036. doi: 10.3390/microorganisms13051036 (PMC12114552; doi:10.3390/microorganisms13051036)

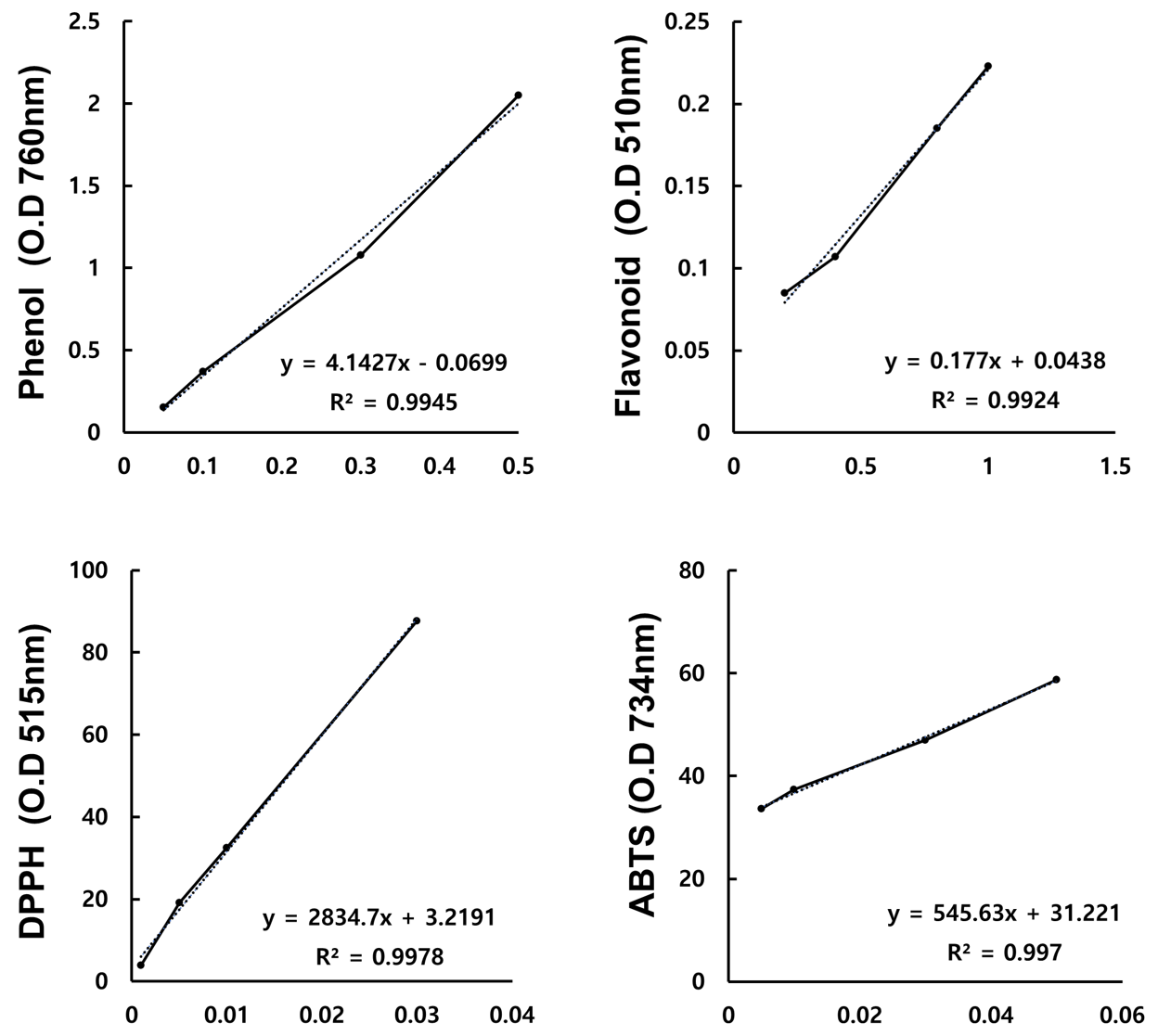

Supplement: Supplementary file 1 [file microorganisms-13-01036-s001.zip › Figure S1.png]

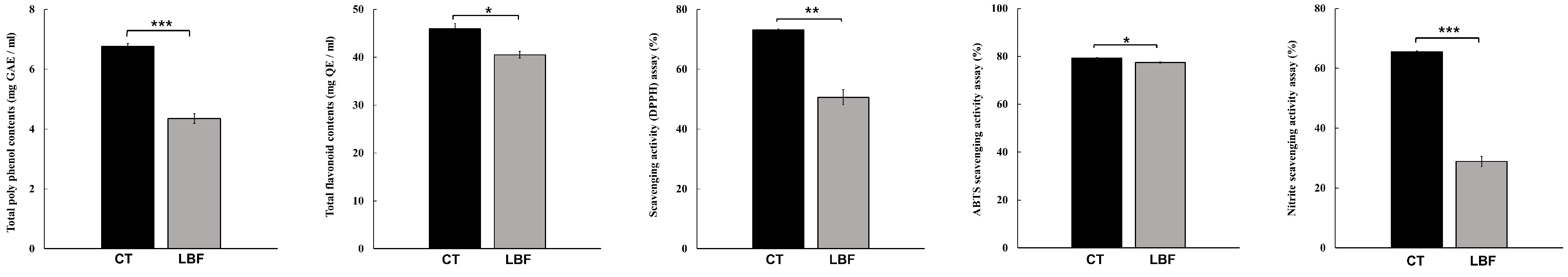

Supplement: Supplementary file 1 [file microorganisms-13-01036-s001.zip › Figure S2.jpg]
